# Microfluidic Production of Mechanochromic Photonic Fibers Containing Nonclose‐Packed Colloidal Arrays

**DOI:** 10.1002/smsc.202000058

**Published:** 2021-02-24

**Authors:** Jong Hyun Kim, Kyung Han Kim, Gun Ho Lee, Ji-Won Kim, Sang Hoon Han, Chang-Soo Lee, Shin-Hyun Kim

**Affiliations:** ^1^ Department of Chemical and Biomolecular Engineering Korea Advanced Institute of Science and Technology (KAIST) Daejeon 34141 Republic of Korea; ^2^ Department of Chemical Engineering and Applied Chemistry Chungnam National University Daejeon 34134 Republic of Korea

**Keywords:** colloidal arrays, mechanochromic materials, microfluidic jetting, photonic fibers, structural colors

## Abstract

Photonic fibers are important raw materials for structurally colored fabrics. In particular, the mechanochromic fibers potentially provide color‐tunable clothes, like chameleon skins. Herein, microfluidic jetting to continuously produce mechanochromic fibers in a controlled manner is used. The jet is produced by flow‐focusing the photocurable dispersions in a surfactant‐laden carrier fluid using microfluidic devices. The dispersions contain monodispersed silica particles in an elastomer‐forming resin. As the silica particles have repulsive interparticle potential in the resin, they spontaneously organize into a nonclose‐packed regular array, developing structural colors. The jet of the dispersions is photocured in situ by irradiation of ultraviolet at the exit of the microfluidic channel, continuously producing the photonic fibers. As the interparticle separation among silica particles is shortened along the radial direction by stretching the elastic fibers, the structural colors are dynamically blue shifted. Also, the original colors are reversibly recovered by relaxing the fibers as the deformation is fully elastic. The nonclose‐packed array of inelastic silica particles provides a wide range of color tuning and high reversibility. Moreover, microfluidic jetting enables the production of Janus fibers composed of two distinct color domains, which enriches the available structural colors through color mixing.

## Graphical abstract

Mechanochromic photonic fibers are continuously produced by microfluidic jetting of photocurable dispersions. The fibers show structural colors that are dynamically and reversibly tunable with mechanical strain. The fibers are further designed to have two distinct domains, which develop mixed colors, enriching the color variety. The photonic fibers are woven to provide mechanochromic fabrics and clothes.

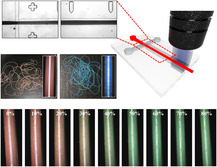

## 1 Introduction

Colloidal arrays with a consistent interparticle distance, in the form of either crystals or glasses, show structural colors through wavelength‐selective diffraction of visible light. As the resonant wavelength for the diffraction is determined by the interparticle distance and refractive index, various colors can be developed using a single set of materials, unlike chemical pigments.^[^ ^1^, ^2^, ^3^, ^4^, ^5^, ^6^, ^7^, ^8^, ^9^, ^10^ ^]^ More importantly, the structural color can be dynamically tuned by changing the interparticle distance or refractive index in real time. For example, interparticle separation can be controlled by applying an electric field on suspensions of charged colloidal particles or composites of colloidal particles or cavities in the matrix of electroresponsive polymers, resulting in structural‐color change.^[^ ^11^, ^12^, ^13^, ^14^ ^]^ Such field‐induced dynamic color changes are promising for displays operated in the reflection mode. On the other hand, the colloidal arrays have been designed to show structural color changes under various conditions, such as humidity, temperature, pH, and specific molecules, by making colloidal arrays with stimuli–responsive hydrogels in the colloids or matrix.^[^ ^3^, ^15^, ^16^, ^17^, ^18^, ^19^, ^20^ ^]^ The stimuli‐sensitive color changes are useful for colorimetric sensors. Mechanical strain also has been used as one of the stimuli to dynamically tune the structural colors. Colloidal arrays in the elastic matrix show dynamic and reversible color change during mechanical deformation as the interparticle distance is simultaneously changed. Such mechanochromic materials are useful as colorimetric strain sensors and user‐interactive anticounterfeiting.^[^ ^21^, ^22^, ^23^, ^24^, ^25^, ^26^ ^]^ Furthermore, the mechanochromic materials in conjunction with microactuator arrays potentially provide color‐tunable fabrics and clothes.^[^ ^27^, ^28^ ^]^

To produce color‐tunable fabrics and clothes, mechanochromic fibers are a prerequisite as raw materials. There have been attempts to produce colloidal arrays in a fiber format.^[^ ^27^, ^28^, ^29^, ^30^, ^31^, ^32^, ^33^, ^34^ ^]^ For example, colloidal particles are self‐assembled on the surface of normal fibers by dip coating or electrophoretic deposition.^[^ ^29^, ^30^, ^31^ ^]^ The use of elastic core fiber and composite cladding of colloidal array in the elastic matrix renders the fiber mechanochromic.^[^ ^27^ ^]^ However, the production is limited to a time‐consuming batch process. Although direct electrospinning of colloidal suspension, without any supporting fibers, achieves continuous production, microscale fibers are stacked over during fabrication, making them difficult to handle as individual fibers.^[^ ^32^ ^]^ The continuous production of mechanochromic fiber has been accomplished by either self‐assembling inelastic core‐elastic shell particles on the surface of elastic fiber through the dip‐coating process or direct extraction of the core–shell particles at a high temperature.^[^ ^28^, ^34^ ^]^ The method provides a practical means to produce mechanochromic fibers. However, the mechanochromic fibers have a limited range of color change. The former does not fully utilize the volume of the fiber for structural coloration although mechanical stability is enhanced by the elastic core, making it difficult to reduce the diameter of the fibers while maintaining high color brightness. Furthermore, the method is restricted to produce single‐colored fibers.

Here, we report a microfluidic strategy to produce mechanochromic fibers in a highly controlled and continuous manner. The elastic photonic fibers are prepared by in situ photocuring of a stable jet of photocurable colloidal inks. To formulate the inks, silica particles are dispersed in an elastomer‐forming resin of poly(ethylene glycol) phenyl ether acrylate (PEGPEA) at a volume fraction as high as 33%. As the PEGPEA molecules form a solvation layer on the surface of silica particles, silica particles show consistent interparticle separation in the resin. A small amount of polydopamine (PDA) nanoparticles is additionally dispersed in the resin to enhance color saturation. To make a stable jet, the ink and carrier fluid of surfactant‐dissolved hexadecane are simultaneously injected into a cross‐junction of a microfluidic device. As the interfacial tension between the ink and carrier fluid is very low, a stable jet is formed for a wide range of flow rates, which flow downstream without significant change of the jet width and interfacial undulation. The jet is continuously exposed to ultraviolet (UV) at the exit of the device for in situ photopolymerization of the resin, which results in the continuous formation of photonic fibers. The photonic fibers show structural colors. More importantly, the color can be dynamically and reversibly tuned by stretching and releasing the fibers as the interparticle distance among silica particles is changed by the deformation. As the silica particles form a nonclose‐packed array, a relatively wide range of color shifts and large yield strain are achieved. In addition, Janus fibers with two different structural colors are produced by forming a Janus jet of two different inks, which enriches the variety of structural colors through color mixing. We weave the mechanochromic fibers and show dynamic color change to demonstrate the potential use of the fibers for color‐tunable fabrics.

## 2 Results and Discussion

### 2.1 Microfluidic Production of Elastic Photonic Fibers

**Figure 1:**
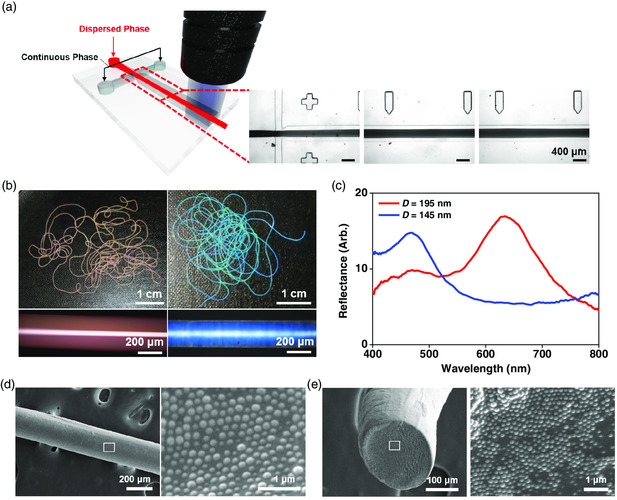
a) Schematics for the continuous production of photonic fibers through in situ photopolymerization of a jet in a microfluidic device and OM images showing the formation of the jet in the cross‐junction and the flow of the jet without interfacial undulation. The second and third OM images are taken at the positions 0.7 and 1.4 cm downstream from the cross‐junction, respectively. b) Sets of photographs and OM images at reflection mode of red‐ (the left panel) and blue‐colored (the right panel) photonic fibers. c) Reflectance spectra of the photonic fibers in (b). d,e) SEM images showing the surface (d) and the cross section (e) of the photonic fibers.

Microfibers have been continuously produced using microfluidic jetting.^[^ ^35^, ^36^, ^37^, ^38^, ^39^ ^]^ Although fluid jets are usually unstable and spontaneously broken to form a series of drops by Plateau–Rayleigh instability, they can be stabilized in a microfluidic channel by confining them with solid walls.^[^ ^40^, ^41^ ^]^ Therefore, microfibers can be produced by in situ solidification of jets using microfluidics. To make the elastic photonic fibers in this work, we produce a jet of photocurable dispersion of silica particles in a carrier fluid in a cross‐junction of the microfluidic device and photopolymerize it in situ by UV irradiation, as shown in **Figure** **1**a. The microfluidic device is prepared by a typical soft lithography technique using poly(dimethylsiloxane) (PDMS), where the channel height is set to 300 μm.

To make elastic photonic structures in the fibers, the photocurable dispersion is formulated by dispensing monodispersed silica particles in PEGPEA at the volume fraction of *ϕ* = 0.33. PEGPEA forms an elastomer when it is polymerized by UV irradiation in the presence of a photoinitiator, whereas it makes a solvation layer on the surface of silica particles through hydrogen bonds, which enables the silica particles to form a nonclose‐packed colloidal array.^[^ ^42^, ^43^ ^]^ Therefore, photopolymerization produces a photonic elastomer containing a nonclose‐packed colloidal array. As a carrier fluid, we use hexadecane containing 4 w/w% surfactant of ABIL EM 90, presaturated by PEGPEA. The presence of the surfactant reduces the interfacial tension between PEGPEA dispersion and hexadecane from 2.22 to 0.62 mN m^−1^, as shown in Figure S1, Supporting Information, which helps stabilize the jet. The saturation of the carrier fluids with PEGPEA is important to prevent the dissolution of PEGPEA of the jet into carrier fluid, which causes the formation of close‐packed particle arrays without polymerized PEGPEA matrix on the surface of the fiber, as shown in Figure S2, Supporting Information.

The photocurable dispersion is injected through the central channel, whereas the carrier fluid is injected through two side channels at the same time. The dispersion forms a jet at the cross‐junction, which flows downstream without interfacial undulation or significant change in the diameter, as shown in Figure 1a. As the interfacial tension is as low as 0.62 mN m^−1^, Plateau–Rayleigh instability is highly suppressed in a wide range of flow rates of the jet and carrier, which enables us to control the diameter of the fiber through the flow‐rate control. When the flow rate of the carrier fluid is increased from 15 to 25 μL min^−1^ while maintaining the flow rate of the jet at 20 μL min^−1^, the jet diameter is reduced from 307 to 208 μm, as shown in Figure S3, Supporting Information. The jets are photopolymerized by continuous irradiation of UV at the exit of the microfluidic device. The in situ photopolymerization does not cause any measurable change in the diameter. For the diameter of 208 μm, the production rate is 66 cm min^−1^. The fiber is continuously produced without any problem at least for 10 min and it is expected that the device can be operated for a much longer timescale.

The photonic fibers show structural colors as the silica particles form a regular array in the fiber. The colors depend on the diameter of silica particles, *D*. With two different sizes of silica particles of *D* = 195 nm and 145 nm, red‐ and blue‐colored fibers are produced, respectively, as shown in Figure 1b. The reflectance spectra of fibers show peaks at *λ*_max_ = 625 nm and 468 nm, respectively, as shown in Figure 1c. The peak positions are roughly consistent with the wavelength of Bragg's diffraction for (111) planes of nonclose‐packed face‐centered‐cubic (fcc) lattice.^[^ ^44^, ^45^ ^]^

(1) $$\lambda_{111}=2d_{111}n_{\text{eff}}={\left(\frac{\pi }{3\sqrt{2}\phi }\right)}^{\frac{1}{3}}{\left(\frac{8}{3}\right)}^{\frac{1}{2}}D{\left(n_{p}^{2}\phi +n_{m}^{2}(1-\phi )\right)}^{\frac{1}{2}}$$

where *d*_111_ is (111) lattice plane spacing and *n*_eff_ is an effective refractive index estimated by Maxwell–Garnett's average of refractive indices of particles and matrix, *n*_p_ and *n*_m_. With *n*_p_ = 1.45 (silica) and *n*_m_ = 1.502 (polymerized PEGPEA), *λ*_111_ is calculated as 619 and 460 nm for *D* = 195 and 145 nm, respectively. Direct observation reveals that the colloidal array formed in the matrix of polymerized PEGPEA is far from the perfect crystalline lattice, as shown in Figure 1d,e. Nevertheless, nonclose‐packed hexagonal arrays of silica particles are predominantly observed on the cylindrical surface and the cross section of the fiber. The fibers show rotation‐independent colors along the cylindrical axis. These indicate that the imperfect fcc or hexagonal‐close‐packed (hcp) lattices are formed in the fiber by aligning the hexagonal array, (111) plane of fcc or (0002) plane of hcp, along the cylindrical surface. Considering that the fcc and hcp have the same maximum volume fraction of 0.74 for close packing, the colloidal array in the polymerized PEGPEA is nonclose‐packed as *ϕ* = 0.33.

### 2.2 Enhancement of Color Saturation

The photonic fibers show structural colors through structural resonance by a regular particle array. At the same time, Mie scattering occurs on the colloidal array and the surface of the fiber, which lowers the color saturation. To enhance the color saturation, we additionally disperse polydopamine (PDA) nanoparticles with an average diameter of 100 nm in the photocurable dispersions of silica particles. PDA nanoparticles absorb a broad range of visible light so that they can enhance the color saturation by suppressing the incoherent scattering while insignificantly affecting the hue.^[^ ^26^ ^]^ However, the concentration of PDA nanoparticles, *C*_PDA_, should be optimized in our system because PDA nanoparticles have no solvation layer, so that they interrupt the formation of a regular array of silica particles.

**Figure 2:**
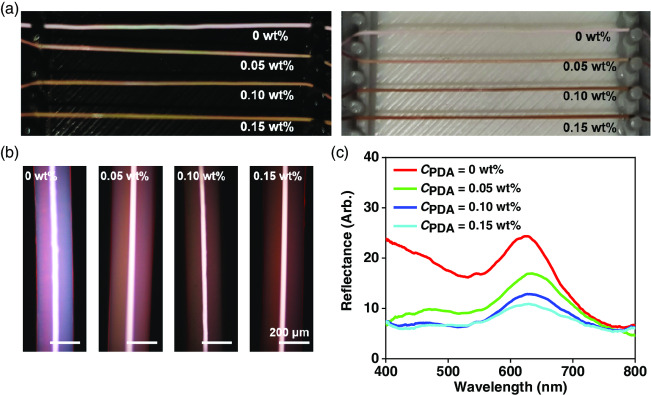
a) Photographs of photonic fibers with four different concentrations of PDA nanoparticles as denoted in black (the left panel) and white (the right panel) backgrounds. b,c) OM images taken at reflection mode and reflectance spectra of photonic fibers in (a).

To investigate the influence of PDA nanoparticles, we vary *C*_PDA_ from 0 to 0.15 w/w% in the photocurable dispersions and produce photonic fibers, as shown in **Figure** **2**a. The colors are compared for the fibers on black and white backgrounds. The color brightness decreases on both backgrounds along with *C*_PDA_ as PDA absorbs visible light, whereas the color saturation is enhanced for *C*_PDA_ = 0.05 w/w% but lowered for *C*_PDA_ = 0.10 w/w% and 0.15 w/w%. This tendency is clearly observed with optical microscopy, as shown in Figure 2b. Without PDA nanoparticles, the fiber is whitish red. For *C*_PDA_ = 0.05 w/w%, the whitening is reduced, developing a dark reddish color with enhanced saturation. Higher *C*_PDA_ renders the fibers darker and no enhancement of color saturation is observed. The reflectance spectra further clarify the change of the color, as shown in Figure 2c. Without PDA nanoparticles, the fiber shows high reflectance at the resonant wavelengths. However, the reflectance intensity at off‐resonant wavelengths is also high, which reduces the color saturation. For *C*_PDA_ = 0.05 w/w%, the reflection at the off‐resonant wavelengths is highly reduced, whereas that at the resonant wavelengths is relatively less reduced, enhancing the color saturation. For higher *C*_PDA_, the reflection at the resonant wavelengths is further reduced, whereas that at the off‐resonant wavelengths is weakly reduced, rendering the color dark and less pronounced. Therefore, we set the optimum concentration for high saturation and brightness as *C*_PDA_ = 0.05 w/w%.

### 2.3 Mechanochromic Property of Photonic Fibers

**Figure 3:**
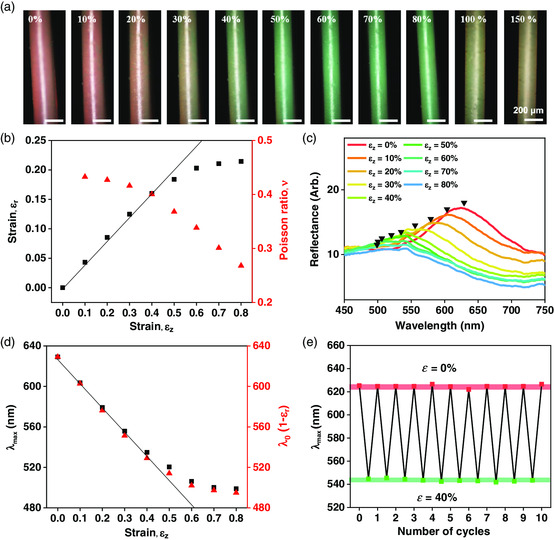
a) Series of OM images of a photonic fiber with various axial strains (*ε*_z_) as denoted. The structural color blue shifts along with the strain up to 80%, which is partially and completely lost at the strains of 100% and 150%, respectively. b) The radial strain (*ε*_r_, the left *y*‐axis) and Poisson ratio (*ν*, the right *y*‐axis) of the fiber as a function of *ε*_z_, where a solid line indicates linearity between *ε*_r_ and *ε*_z_ for *ε*_z_ < 40%. c) Reflectance spectra of the fiber for various axial strains, as denoted. The stopband positions are denoted with inverted triangles. d) The reflectance peak position (*λ*_max_, the left *y*‐axis) and the resonant wavelength estimated from *ε*_r_ (the right *y*‐axis), where a solid line indicates linearity between *λ*_max_ and *ε*_z_ for *ε*_z_ < 40%. e) The shift of *λ*_max_ for ten cycles of stretching to *ε*_z_ = 40% and relaxation to *ε*_z_ = 0.

The photonic fibers contain a nonclose‐packed array of inelastic silica particles in an elastic pPEGPEA matrix. Assuming a perfect fcc lattice, the surface‐to‐surface separation between two neighboring silica particles is estimated as 60 nm for *D* = 195 nm and *ϕ* = 0.33, see Figure S4, Supporting Information. Although the separation is small, it is expected that the separation facilitates the deformation of the structure without rearrangement of the inelastic silica particles in comparison with a close‐packed array.^[^ ^24^ ^]^ Therefore, the photonic fibers can show a reversible change of structural colors for an enhanced range of stretching and relaxation. To study the mechanochromic property, we use the fiber composed of silica particles with *D* = 195 nm at *ϕ* = 0.33 and *C*_PDA_ = 0.05 w/w%. Upon stretching up to the extensional strain along the axis of *ε*_z_ = 80%, the structural color blue shifts from dark red to green while the fiber is slender, as shown in **Figure** **3**a. The blue shift and recovery of the structural color during stretching and relaxation are observable with naked eyes, as shown in Movie S1, Supporting Information. When the fiber is further stretched, the structural color is faint at *ε*_z_ = 100% and completely disappears at *ε*_z_ = 150%; the native brown color of PDA nanoparticles is disclosed at *ε*_z_ = 150%. The fibers are stretchable up to *ε*_z_ = 200% and broken at larger strain.

The blue shift of the structural color along with the extensional strain is caused by the reduction of interparticle separation along the radial direction of the fiber. That is

(2) $$\lambda_{\text{max}}(\varepsilon_{z})=\lambda_{0}(1-\varepsilon_{r})=\lambda_{0}(1-\text{ν}\varepsilon_{z})$$

where *λ*_0_ is the resonant wavelength for *ε*_z_ = 0, *ε*_r_ is the strain along the radial direction, and *ν* is Poisson's ratio. The value of *ε*_r_ can be directly measured as a function of *ε*_z_ from the optical microscope (OM) images of the fiber in Figure 3a and the value of *ν* is calculated from the slope, as shown in Figure 3b. The value of *ν* is larger than 0.4 for *ε*_z_ < 40%, which is close to the value for rubber (0.5), indicating that the pPEGPEA rubber matrix deforms with a negligible influence of inelastic silica particles as expected from the nonclose‐packed array. The value of *ν* rapidly decreases from 0.40 to 0.27 as *ε*_z_ increases from 40% to 80%, where the inelastic silica particles get closer, resisting the deformation of the elastic matrix.

The blue shift of the structural color can be quantitatively measured using reflectance spectra, as shown in Figure 3c. At *ε*_z_ = 0, the reflectance spectrum has a peak at *λ*_max_ = 629 nm. As *ε*_z_ increases to 80%, *λ*_max_ gradually shifts to *λ*_max_ = 499 nm, as shown in Figure 3d. The shift is linear for *ε*_z_ < 40%, and slows down for *ε*_z_ > 40%. This tendency is in good agreement with *λ*_max_(*ε*_z_) calculated using Equation (2) from *ε*_r_. That is, the loss of linearity for *ε*_z_ > 40% originates from the deformation resistance caused by a packing of inelastic silica particles with reduced interparticle separation. The surface‐to‐surface separation between two closest neighbors at *ε*_z_ = 40% is estimated as 34 nm by assuming that the silica particles in a perfect fcc lattice move by following the deformation of the pPEGPEA matrix with *ε*_r_ = 16% (see Figure S4, Supporting Information), which is much reduced from 60 nm at *ε*_z_ = 0. During the blue shift, the reflectivity at the resonant wavelengths gradually decreases. This is because the reduction of the interparticle separation along the radial direction leads to the decrease in refractive index contrast between the particle‐rich region and particle‐poor region.^[^ ^26^ ^]^ The reduction of structural resonance makes the structural color overwhelmed by the native brown color of PDA nanoparticles for *ε*_z_ > 80%, as shown in Figure 3a.

The color change is reversible, as shown in Figure 3e. When the fiber is stretched to *ε*_z_ = 40% and relaxed to *ε*_z_ = 0 for ten cycles, the resonant wavelengths at two states remain unchanged at *λ*_max_ = 543 and 625 nm, respectively; the maximum deviations from the average values are ≈2 nm (0.4% deviation) and 3 nm (0.1% deviation), respectively. Even for the same cycle test with *ε*_z_ as high as 150%, the original resonant wavelength and reflectance intensity are fully recovered without any hysteresis, as shown in Figure S5, Supporting Information. The maximum deviation from the average value is 0.57 (3.3% deviation) and there is no trend of the change along with the cycle number.

The photonic fiber composed of silica particles with *D* = 145 nm at *ϕ* = 0.33 and *C*_PDA_ = 0.05 w/w% shows a blue color and the resonant wavelength at *λ*_max_ = 450 nm without stretching, as shown in Figure S6, Supporting Information. When the fiber is stretched, the color gradually turns violet and faint without a dramatic color change as the resonant wavelength blue shifts from *λ*_max_ = 450 nm toward the UV region. The color shift and discoloration during stretching and the recovery and relaxation are shown in Movie S2, Supporting Information. This change is also highly reversible and no hysteresis is observed.

### 2.4 Mechanochromic Property of Woven Fabrics

**Figure 4:**
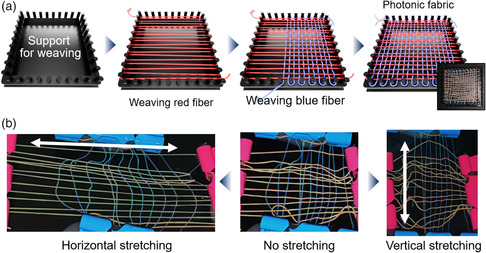
a) Schematics showing the procedure to horizontally and vertically weave red‐ and blue‐colored fibers using square‐shaped support, respectively, and a photograph of the woven fabric. b) Photographs showing the color changes for horizontal stretching (the left panel) and vertical stretching (the right panel) from the original fabric (the middle panel).

The red‐ and blue‐colored photonic fibers are woven to make a mechanochromic fabric. To render the fabric to show anisotropic color change depending on the direction of stretching, the red and blue photonic fibers are horizontally and vertically woven, respectively. To help weaving, square‐shaped support is prepared by 3D printing to have ten posts along each edge of the square, as shown in **Figure** **4**a. The red‐colored fiber is repeatedly wound using the pairs of ten posts in the left and right edges along the horizontal direction. Afterward, the blue‐colored fiber is vertically woven along the horizontally aligned red fiber using a needle, where the pairs of ten posts in the top and bottom edges are used to guide weaving. The resulting fabric can be released from the support although it is loosely woven for this demonstrative purpose. To show the anisotropic color change, the fabric is held using four clips and stretched. When the fabric is stretched horizontally, the red fibers turn green whereas the blue fibers remain unchanged, as shown in the left panel of Figure 4b. In contrast, when the fabric is stretched vertically, the blue fibers turn faint, whereas the red fibers remain unchanged, as shown in the right panel. The anisotropic color change is shown in Movie S3, Supporting Information.

### 2.5 Production of Janus Photonic Fibers

**Figure 5:**
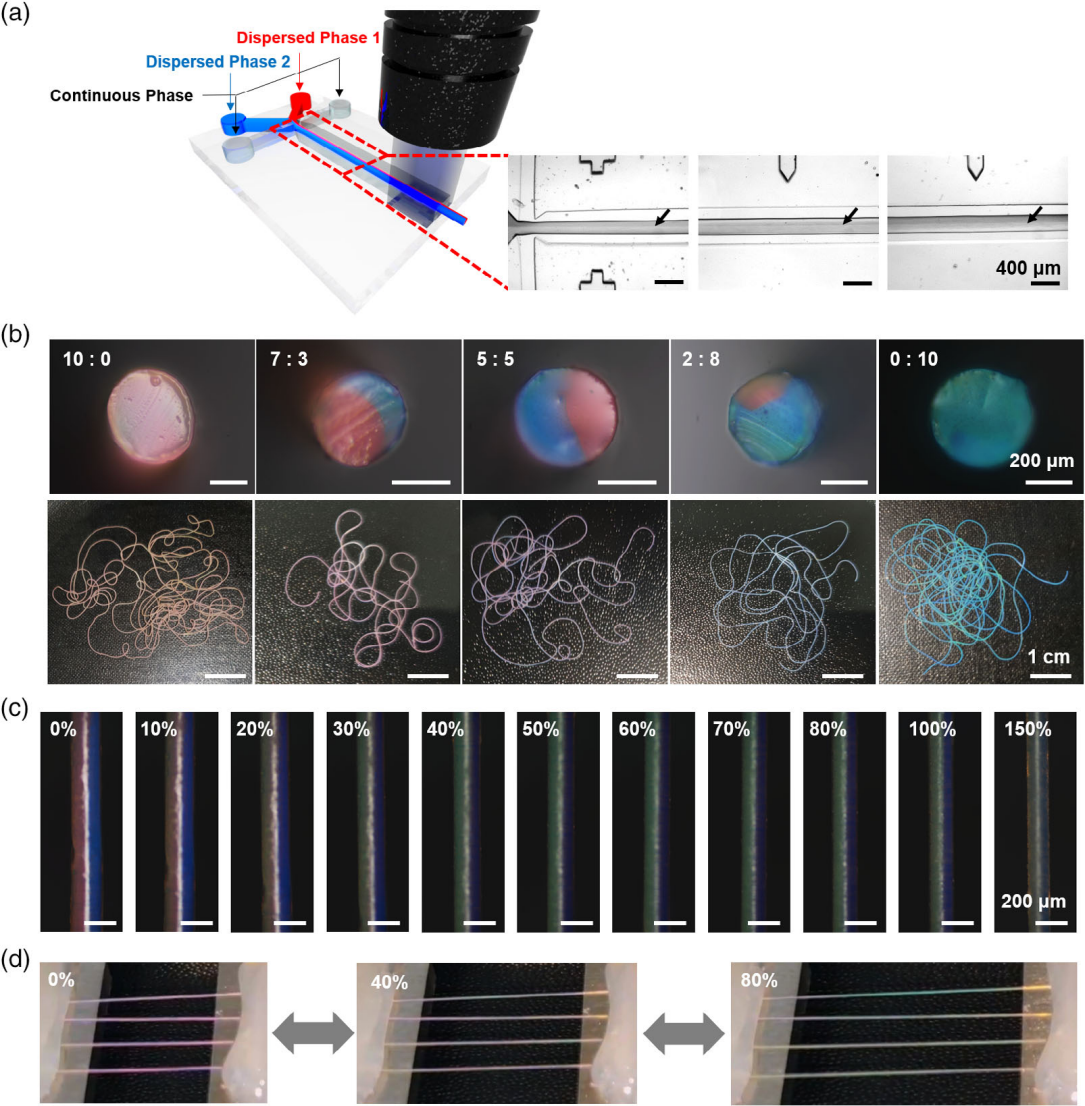
a) Schematics for the microfluidic production of Janus photonic fibers and OM images showing the formation and flow of the Janus jet, where the arrows indicate the boundary between two distinct dispersions. b) Sets of OM images showing the cross section and photograph of Janus fibers with various fractions of red‐to‐blue domains, as denoted. The Janus fibers show mixed colors depending on the ratio in the photographs. c,d) OM images and photographs of the 5:5 Janus photonic fiber with various axial strains as denoted. The photonic fiber shows magenta at *ε*_z_ = 0, which turns to cyan at *ε*_z_ = 80% through color mixing in (d).

Microfluidic jetting enables the production of Janus fibers by forming the jet with two distinct solutions.^[^ ^36^ ^]^ We produce Janus photonic fibers using two distinct dispersions of silica particles, which contain silica particles with *D* = 195 and 145 nm, respectively. To inject the dispersions at the same time and form a Janus jet in a carrier fluid, we use a microfluidic device that has a “Y”‐shaped injection channel, as shown in **Figure** **5**a. The two dispersions form a single Janus jet at the cross‐junction, which flows downstream while maintaining the boundary between two dispersions, as indicated by arrows. Therefore, in situ photopolymerization produces Janus photonic fibers, as shown in Figure 5b. The Janus fibers show red and blue in their own domains composed of silica particles with *D* = 195 and 145 nm, respectively.

The relative fraction of the two domains is adjustable by controlling the relative flow rate of two dispersions, as shown in Figure 5b. Two domains with red and blue colors are clearly observed in the cross sections of fibers under optical microscopy, as shown in the top panels. When observed with naked eyes that two different colors are not distinguished. Instead, the fibers show mixed colors depending on the relative ratio of two domains, as shown in the bottom panels. As the fraction of the blue domain increases from 0 to 100%, the color of the fibers gradually changes from red to magenta and blue. Therefore, the Janus fibers can expand the available structural colors through color mixing.

The Janus photonic fibers also show a reversible mechanochromic property. As the photonic fiber with a ratio of 5:5 for red and blue domains is stretched to *ε*_z_ = 80%, the red domain turns green whereas the blue domain turns violet, as shown in Figure 5c. When the fiber is further stretched to *ε*_z_ = 100% and 150%, the structural colors turn faint and almost disappear. The independent color changes of two domains in the Janus fiber develop the change of mixed color when observed with naked eyes, as shown in Figure 5d and Movie S4, Supporting Information. The fibers show magenta color due to the color mixing of red and blue without stretching, which turn cyan as they are stretched because red turns green and blue turns violet.

## 3 Conclusion

In this work, we continuously produce elastic photonic fibers using microfluidic jetting and characterize their mechanochromic property. The jets are produced in a surfactant‐laden carrier fluid by flow‐focusing the photocurable dispersions of silica particles in an elastomer‐forming resin of PEGPEA in a cross‐junction of microfluidic devices. In situ photopolymerization of the photocurable jets at the exit of the microfluidic channel results in the continuous production of fibers. The diameter of the fibers is adjustable through the control of flow rate in a certain range, which can be significantly increased or decreased using the device with the same design but different dimensions. In addition, it is potentially possible to produce fibers with a noncircular cross section through flow control.^[^ ^46^ ^]^ In the fibers, the silica particles spontaneously form a nonclose‐packed regular array in the resin, developing structural colors. More importantly, the elastic pPEGPEA matrix enables the structural deformation of the regular array so that the structural colors are dynamically tunable. The nonclose‐packed structure facilitates deformation without a rearrangement of inelastic silica particles, providing a relatively wide range of color tuning and high yield strain; the fabrication method, colloidal packing state, and mechanochromic performance of the fibers are compared with previously reported fibers based on colloidal self‐assembly in Table S1, Supporting Information. By parallelly injecting two distinct dispersions into the microfluidic devices, a Janus jet can be formed, which turns into a Janus photonic fiber. The Janus fibers possess two different structural colors, which show a mixed color when observed by naked eyes. Therefore, the Janus configuration enriches the variety of structural colors. In this early stage of development, we simply demonstrate the anisotropic color change of loosely woven fabrics composed of red‐ and blue‐colored fibers that are aligned perpendicularly. As the fibers are mechanically stable and have a large yield strain, they can be potentially further woven to complicated fabrics using an elaborate knitting machine.^[^ ^28^ ^]^ We believe that the mechanochromic fibers can be further developed to improve color brightness and mechanochromic performance. For example, the reflectivity at the stopband position and the linearity between the stopband position and the strain can be conserved during mechanochromic changes by causing larger interparticle separation. Although the solvation layer‐induced repulsion is effective in the separation of ≈60 nm so that it is unable to make the larger separation, other mechanisms of interparticle repulsion can be used to make nonclose‐packed arrays with sufficient separation. The mechanochromic fibers with improved performance are potentially useful as raw materials to design color‐tunable clothes.

## 4 Experimental Section

4.1

4.1.1

##### Preparation of Colloidal Dispersions

Monodispersed silica particles with diameters of 145 and 195 nm (Sukgyung AT) were respectively washed out with ethanol and fully dried in a 70 °C convection oven for 3 h. About 1.5 g of silica powder was dispersed in 15 ml ethanol by ultrasonication for 6 h, at which 1.716 g of PEGPEA (*M*_n_ 324, Sigma‐Aldrich) containing 1 w/w% photoinitiator of 2‐hydroxy‐2methyl‐1phenyl‐1‐propanone (Darocur 1173, Ciba chemical) was added and mixed by additional ultrasonication. PDA nanoparticles with a diameter of 100 nm, synthesized by the oxidative polymerization of dopamine hydrochloride,^[^ ^47^ ^]^ were dispersed into the silica–PEGPEA–ethanol dispersion. PDA nanoparticles were originally synthesized in water, which were transferred to ethanol before being added to silica dispersion through several times of centrifugation at 12 000 rpm and ultrasonication. The concentration of PDA nanoparticles was set to 0.05, 0.1, and 0.15 w/w% in ethanol‐free basis, respectively. Ethanol was fully dried in a 70 °C convection oven for 18 h and the dispersions were mechanically agitated using a planetary centrifugal bubble‐free mixer (AR‐310, Thinky) before use.

##### Production of Mechanochromic Fiber

PDMS microfluidic devices with a cross‐junction geometry were prepared by a standard soft lithography technique. A master mold was fabricated by typical photolithography using a SU‐8 photoresist on a silicon wafer. A mixture of PDMS prepolymer and curing agent in a mass ratio of 10:1 (Sylgard 184, Dow Corning Co.) was stirred using a planetary centrifugal bubble‐free mixer (AR‐310, Thinky) and degassed in a vacuum desiccator. The PDMS mixture was poured onto the master mold and cured at 65 ° for 12 h in a convection oven. After curing, the PDMS was peeled off from the mold, which was punched to make holes for the inlets. The PDMS was exposed to oxygen plasma for 2 min and bonded to a PDMS substrate. The outlet was formed by cutting the PDMS device at the channel downstream with a razor blade. The microfluidic channel had dimensions of 200 × 300 μm (width × height) in the injection area and 450 × 300 μm in the jet‐formation area. The silica–PDA–PEGPEA dispersion and PEGPEA‐saturated hexadecane (99%, Sigma‐Aldrich) containing 4 w/w% ABIL EM 90 (Evonik) were simultaneously injected into the device using syringe pumps (PHD ULTRA, Harvard Apparatus). Typical flow rates of dispersion and hexadecane were 20 and 25 μL min^−1^, respectively. The dispersion formed a jet at the cross‐junction, which flowed downstream. As the devices were operated in a glass petri dish containing the continuous phases, the jet flowed out from the device, which was continuously irradiated with UV (LPF100, LIIM Tech.) with the intensity of 6 W cm^−2^ at the exit; the entire surfaces of the devices were wrapped using a black tape to prevent the photopolymerization of PEGPEA in the device, except the exit. The fibers were further cured by UV irradiation for 30 s after the collection.

##### Characterization

The jet formation in the microfluidic devices was monitored with inverted optical microscopy (Eclipse TE2000‐U, Nikon). The resulting fibers were observed using optical microscopy at reflection mode (Eclipse L150, Nikon) and reflectance spectra were measured using a fiber‐coupled spectrometer (HR400CG‐UV‐NIR, Ocean Optics, Inc.). The surface and cross‐sectional images of the fibers were observed using a scanning electron microscope (SEM) (Magellan400, FEI company) after OsO_4_ coating. 3D molds were printed by a fused deposition modeling (FDM)‐type 3D printer (3DWOX DP200, Sindoh Co., Ltd.) and used to weave the fibers.

## Conflict of Interest

The authors declare no conflict of interest.

## Data Availability Statement

Research data are not shared.

## Supporting information

Supplementary Material

Supplementary Material

Supplementary Material

Supplementary Material

Supplementary Material
