# Supplementary material for: Microfluidic Production of Mechanochromic Photonic Fibers Containing Nonclose‐Packed Colloidal Arrays
Source: Small Sci. 2021 Feb 24;1(4):2000058. doi: 10.1002/smsc.202000058 (PMC11935952; doi:10.1002/smsc.202000058)
Supplement: Supplementary file 1 — Supplementary Material [file SMSC-1-2000058-s001.docx]

Copyright WILEY-VCH Verlag GmbH & Co. KGaA, 69469 Weinheim, Germany, 2021.

Supporting Information

**Microfluidic Production of Mechanochromic Photonic Fibers containing Non-close-packed Colloidal Arrays**

Jong Hyun Kim, Kyung Han Kim, Gun Ho Lee, Ji-Won Kim, Sang Hoon Han, Chang-Soo Lee*, and Shin-Hyun Kim*

Department of Chemical and Biomolecular Engineering, Korea Advanced Institute of Science and Technology (KAIST)

Department of Chemical Engineering and Applied Chemistry, Chungnam National University

E-mail: Shin-Hyun Kim ([kim.sh@kaist.ac.kr](mailto:kim.sh@kaist.ac.kr)) and Chang-Soo Lee ([rhadum@cnu.ac.kr](mailto:rhadum@cnu.ac.kr))

**Contents**

**S1. Measurement of interfacial tensions**

**S2. Influence of PEGPEA in carrier fluid**

**S3. Control of the fiber diameter**

**S4. Surface-to-surface separation between two nearest particles**

**S5. Elastic deformation of photonic fibers**

**S6. Mechanochoromic property of blue photonic fibers**

**S7. Comparison of mechanochromic performance with previous works**

**S8. Description for movies**

**S1. Measurement of interfacial tensions**

The interfacial tensions are measured using a pendant drop method for PEGPEA-to-hexadecane interfaces without and with 4 w/w% surfactants of ABIL EM 90 in the hexadecane. A pendant drop of heavier PEGPEA is produced in the hexadecane and the interfacial tension is estimated from the shape of the drop, as shown in Figure S1a. With the densities of PEGPEA and hexadecane, $\rho_{PEGPEA}=1.127 g/{cm}^{3}$and $\rho_{hexadecane}=0.773 g/{cm}^{3}$, the interfacial tension is measured as 2.22 mN m^-1^ for the PEGPEA-to-hexadecane without the surfactant and that is 0.62 mN m^-1^ for the PEGPEA-to-hexadecane with the surfactant.


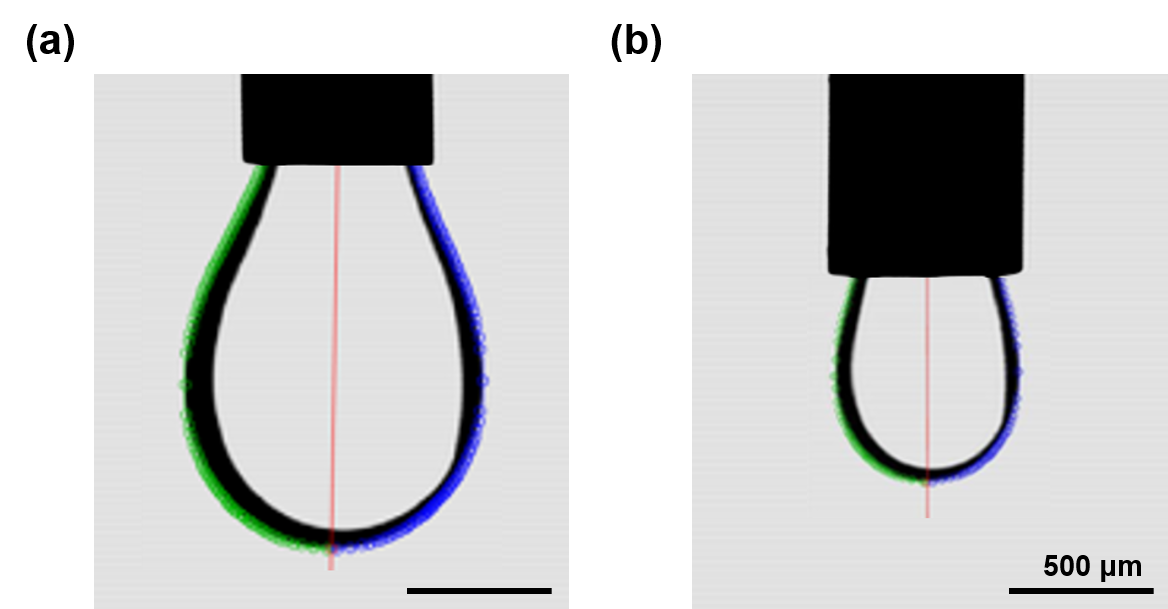


**Figure S1.** (a, b) Optical images showing pendant drops of PEGPEA in hexadecane (a) and surfactant (4 w/w% of ABIL EM 90)-dissolved hexadecane (b).

**S2. Influence of PEGPEA in a carrier fluid**

PEGPEA is partially soluble in hexadecane so that the use of hexadecane without PEGPEA as a carrier fluid results in the dissolution of PEGPEA from the jet. Therefore, silica particles which are not dispensible in the hexadecane form a close-packed array without a PEGPEA matrix along the surface of the fiber as shown in Figure S2, whereas they form a non-close-packed array in the inner part. The close-packed array makes a brittle blue shell on the surface of the fiber, whereas the non-close-packed array makes an elastic red core, as shown in Figure S2a. To avoid the formation of a close-packed array, the hexadecane is pre-saturated with PEGPEA, which prevents the dissolution of PEGPEA to the carrier fluid during the jet formation and flow, as confirmed in Figure 1b and 1c.


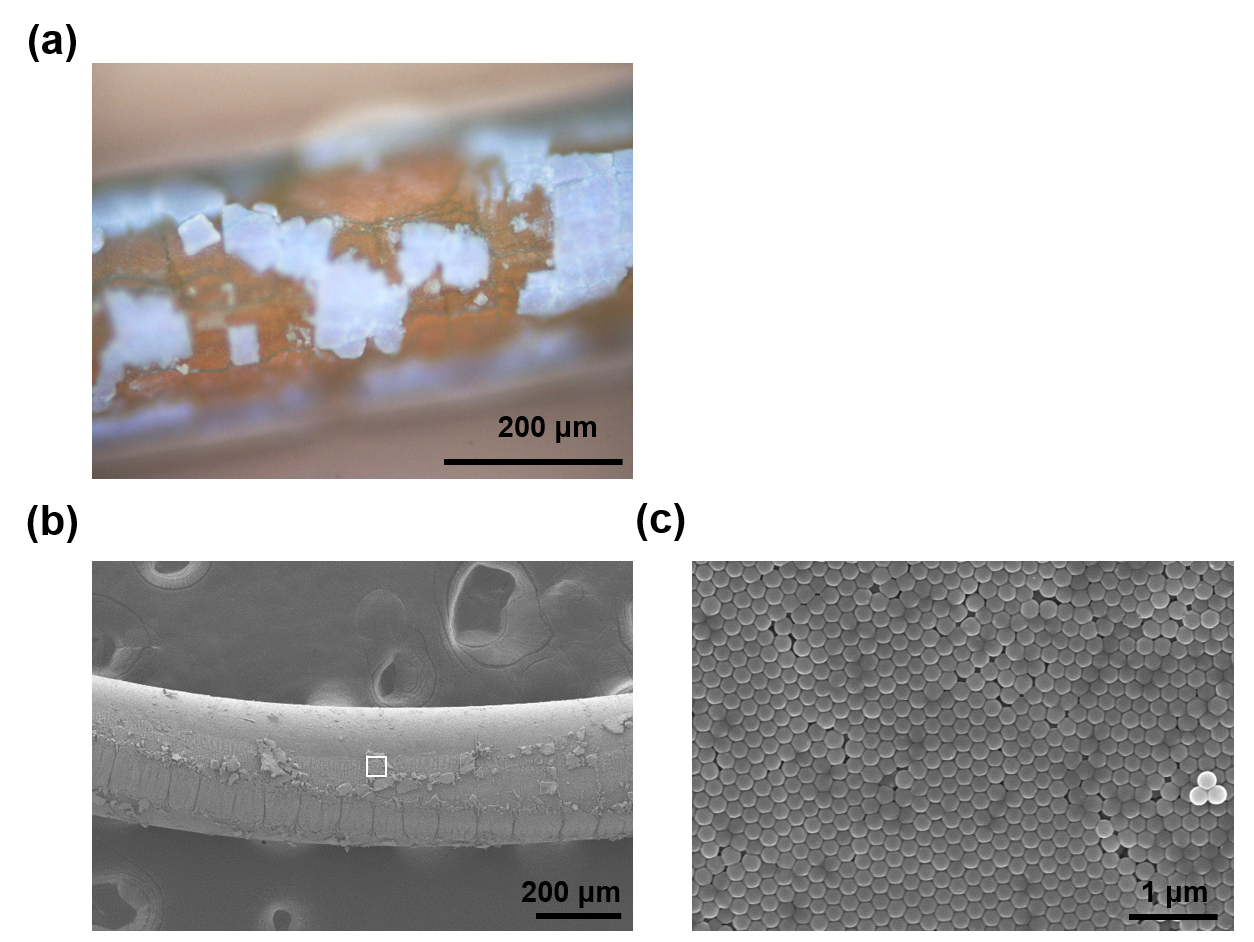


**Figure S2.** (a-c) OM and SEM images of the fibers produced in surfactant-dissolved hexadecane without PEGPEA.

**S3. Control of the fiber diameter**

The diameter of the jet, or fiber, can be controlled by adjusting the flow rate of the carrier fluid during the microfluidic operation. When the flow rate of the carrier fluid is increased from 15 μL min^-1^ to 20 μL min^-1^ and 25 μL min^-1^ while maintaining the flow rate of the jet at 20 μL min^-1^, the average jet diameter is reduced from 307 μm, 247 μm, and 208 μm, as shown in Figure S3; the average diameter is calculated from the diameters at 100 different positions measured using OM image at transmission mode.


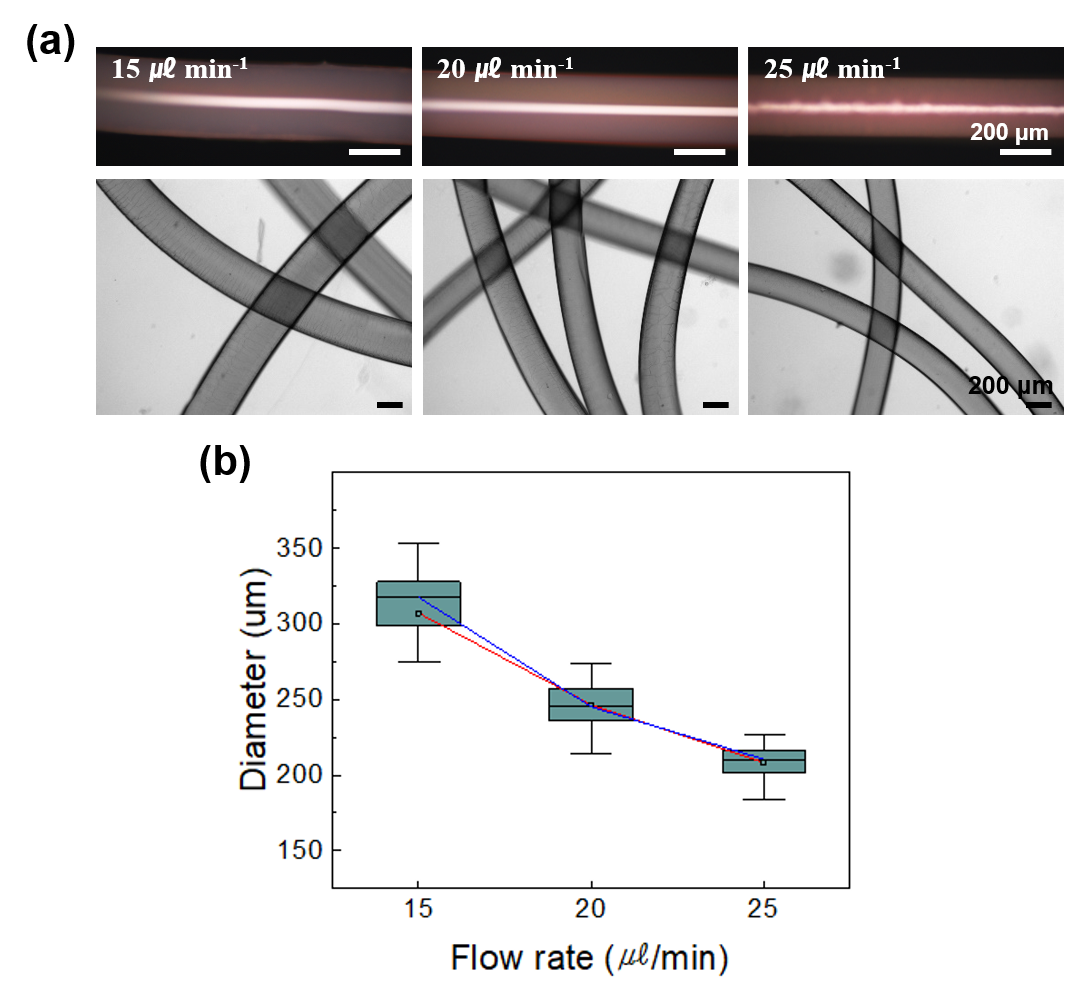


**Figure S3.** (a) Sets of OM image at reflection mode (the top panels) and transmission mode (the bottom panels) of fibers produced with the flow rates of carrier fluid, as denoted. (b) The average diameter of fibers as a function of the flow rate of the carrier fluid.

**S4. The surface-to-surface separation between two nearest particles**

For the non-close-packed fcc structure, the volume fraction of spheres in a unit cell, *ϕ*, can be calculated as:

$\phi= \frac{4\times\frac{\pi}{6}D^{3}}{a^{3}}$, (1)

where *D* is the diameter of spheres and *a* is lattice constant. The center-to-center distance between two nearest neighbors, *d*_cc_, is the same as $a/\sqrt{2}$ and the surface-to-surface separation is 2*t* = *d*_cc_ – *D*, as shown in Figure S4a, b. For the non-close-packed fcc structure with *D* = 195 nm and *ϕ* = 0.33, 2*t* is calculated as 60 nm. The surface-to-surface separation between two nearest neighbors decreases as the fcc lattice stretched along the [111] direction as the particles in two neighboring (111) planes approach each other. At *ε*_z_ = 40%, *ε*_r_ is measured as 16%, as shown in Figure 3b. With *ε*_z_ = 40% and *ε*_r_ = 16%, the lattice can be constructed by moving the particles according to the strains, as shown in Figure S4d. The surface-to-surface separation is calculated as 2*t* = 34 nm for the deformed lattice at *ε*_z_ = 40%.


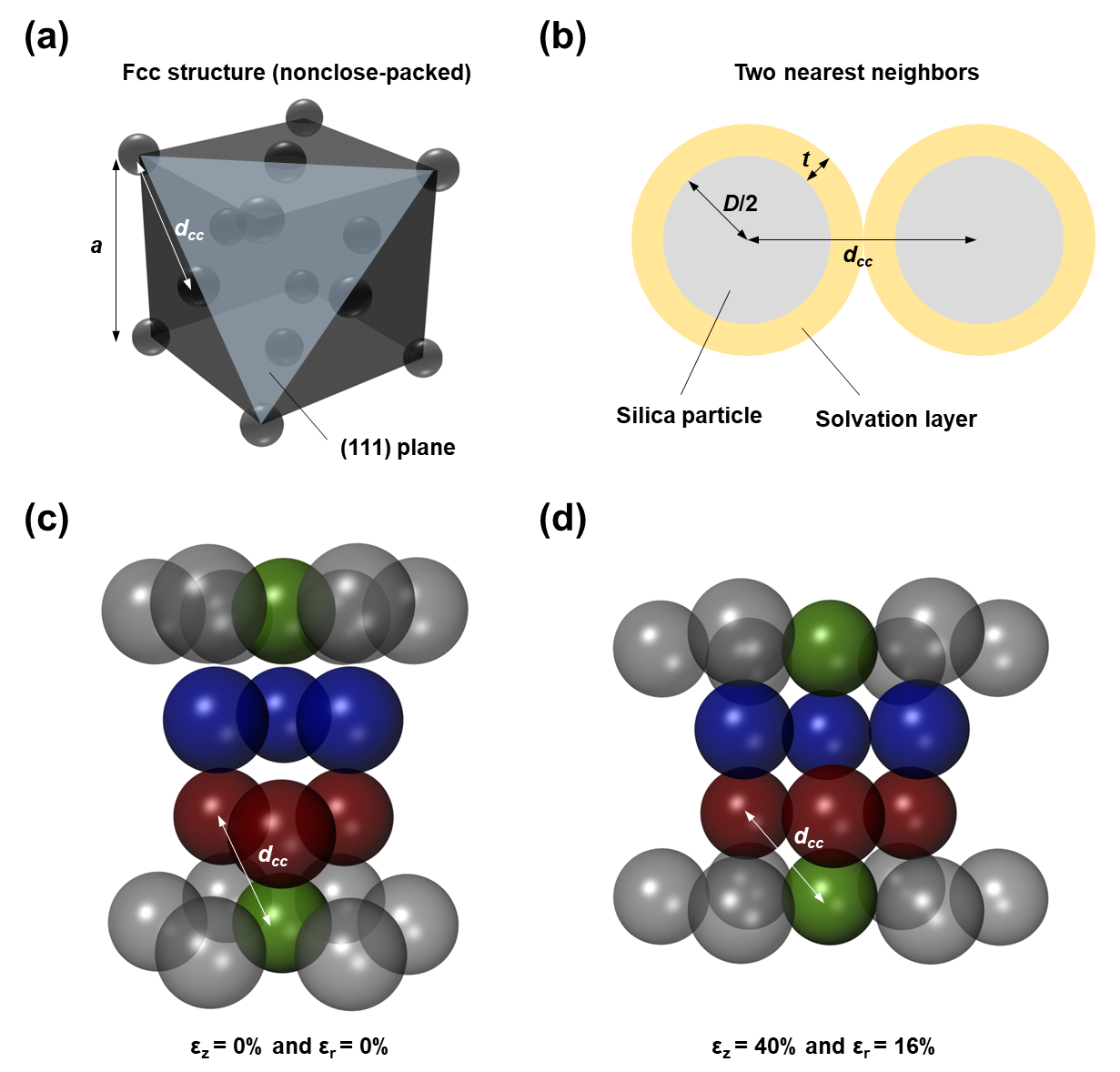


**Figure S4.** (a) The unit cell of non-close-packed fcc lattice. (b) A cartoon showing two nearest neighbors separated by solvation layers. (c, d) Lattice models for the non-close-packed fcc (c) and deformed structure with *ε*_z_ = 40% and *ε*_r_ = 16% (d).

**S5. Elastic deformation of photonic fibers**

To investigate the reversibility of the deformation, the photonic fiber is stretched to have *ε*_z_ = 150% and relaxed to *ε*_z_ = 0 for 10 cycles. As the fiber loses the structural color for *ε*_z_ = 150%, the reflectivity at the resonant wavelength of undeformed fiber is repeatedly measured, as shown in Figure S5; the fiber has a stopband at 629 nm at *ε*_z_ = 0. Even though the strain is as high as 150%, the reflectivity is fully recovered without any hysteresis. The maximum deviation from the average value is 0.57, which corresponds to a 3.3% deviation. Also, there is no trend of the change along with the cycle number, indicating that the deformation is fully elastic. Therefore, it is expected that the yield strain is higher than 150%.

**
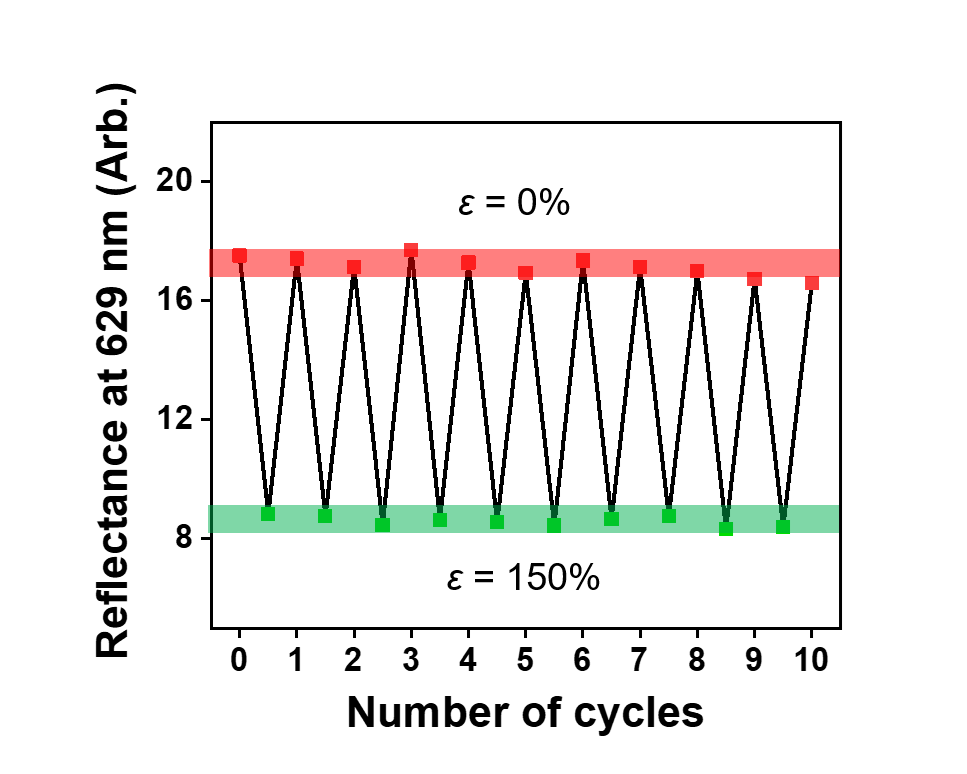
**

**Figure S5.** The change of reflectance intensity at the resonant wavelength of 629 nm at *ε*_z_ = 0 for 10 cycles of stretching to *ε*_z_ = 150% and relaxation to *ε*_z_ = 0.

**S6. Mechanochoromic property of blue photonic fibers**

The blue photonic fibers are produced using the photocurable dispersion of silica particles with *D* = 145 nm at *ϕ* = 0.33 and *C*_PDA_ = 0.05 w/w% in PEGPEA. When the fiber is stretched, the color blue-shifts from blue to violet for *ε*_z_ < 40% and gets fainted for *ε*_z_ > 40% as the resonant wavelength shifts from visible to UV region.


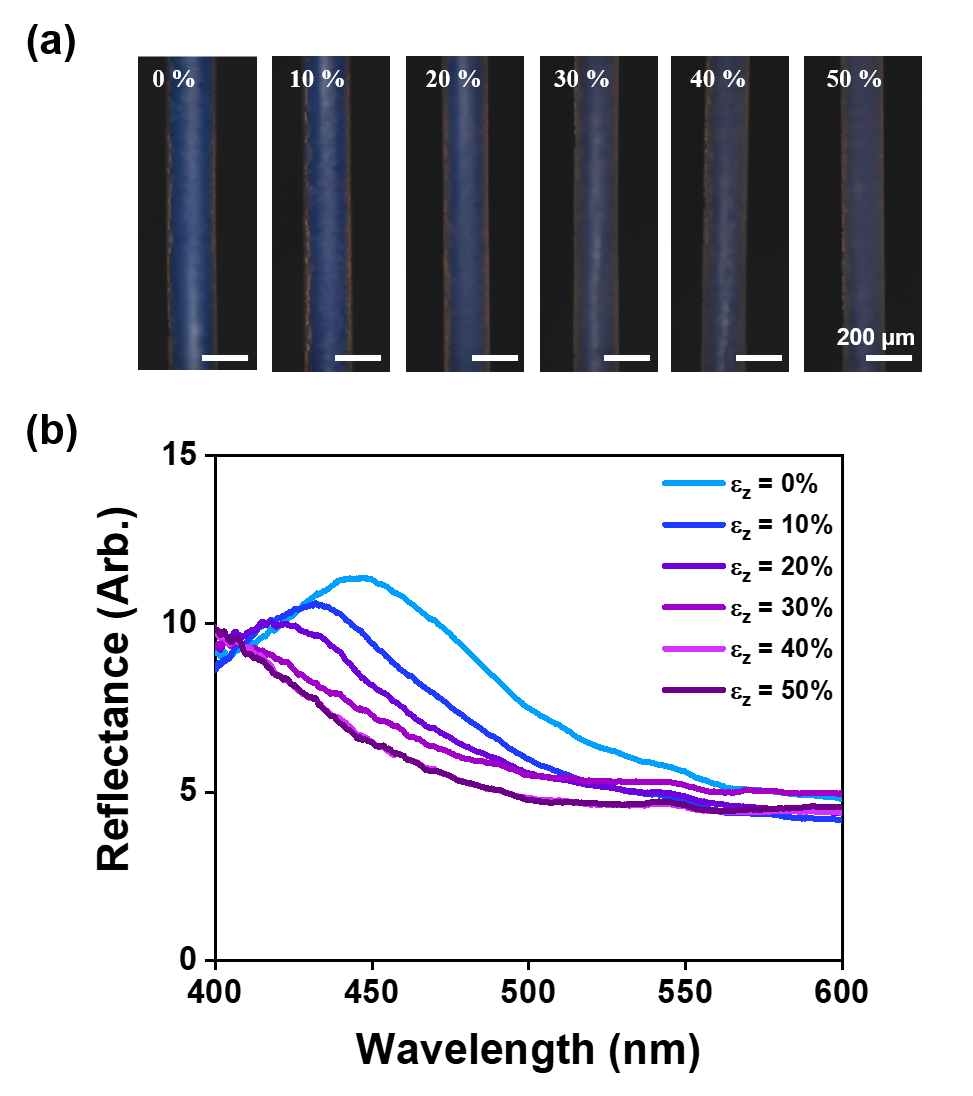


**Figure S6.** (a, b) Series of OM images and reflectance spectra of a photonic fiber with various axial strains (*ε*_z_), as denoted.

**S7. Comparison of mechanochromic performance with previous works**

There are some previous reports on the production of mechanochromic photonic fibers containing colloidal arrays through various techniques. In Table 1, we summarize the type of the production methods, packing state of colloidal particles, the diameter of fibers, maximum strain, maximum peak shift, and reversibility. In all previous works, the colloidal particles are closely packed, which restricts the rearrangement of particles, thus achieving a relatively small degree of color shift. In this work, we improve the color shift by making a non-close-packed colloidal array inside the fibers. The maximum strain is as high as 150% and the maximum shift of stopband is as high as 130 nm, as shown in Table S1.


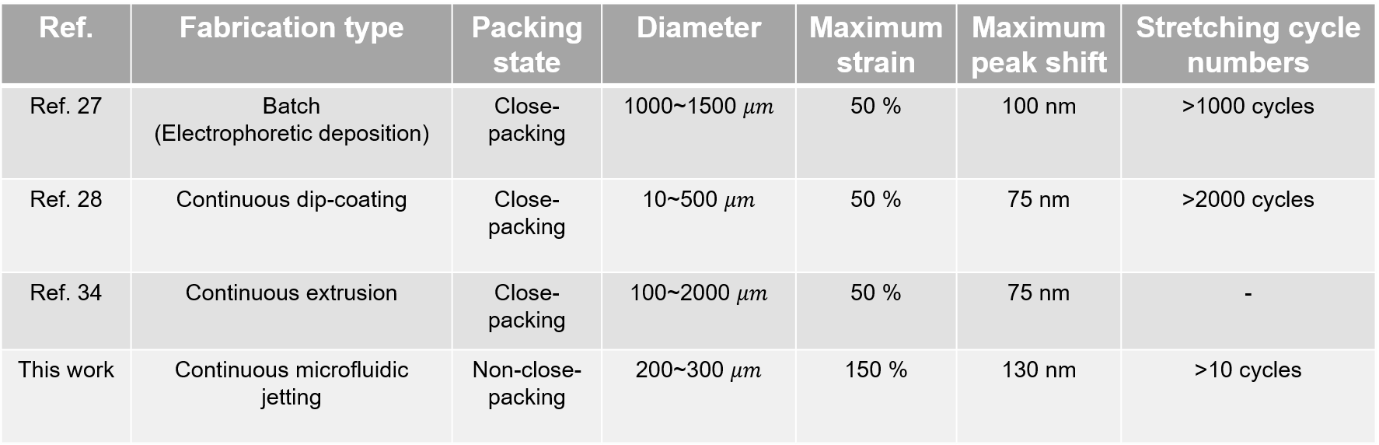


**Table S1.** Summary of the type of the production methods, packing state of colloidal particles, the diameter of fibers, maximum strain, maximum peak shift, and reversibility (cycle test) for previous works and this work.

**S8. Description for movies**

- **Movie S1** shows the reversible color change of red photonic fibers by stretching and relaxation. The movie is 4 times faster than real-time motion.
- **Movie S2** shows the reversible color change of blue photonic fibers by stretching and relaxation. The movie is 4 times faster than real-time motion.
- **Movie S3** shows the anisotropic color change of woven fabric by horizontal and vertical stretching and relaxation. The first half of the movie is for horizontal stretching of red-colored fibers and the second half is for vertical stretching of blue-colored fibers. The fabrics show anisotropic color change. The movie is 4 times faster than real-time motion.
- **Movie S4** shows the reversible color change of Janus photonic fibers by stretching and relaxation. The fibers show magenta color without stretching, which turn cyan as the fibers are stretched. The color returns to magenta color by relaxation. The movie is 4 times faster than real-time motion.
